# Using Collaborative Models to Overcome Obstacles to Undergraduate Publication in Cognitive Neuroscience

**DOI:** 10.3389/fpsyg.2019.00549

**Published:** 2019-03-21

**Authors:** Cindy M. Bukach, Kendall Stewart, Jane W. Couperus, Catherine L. Reed

**Affiliations:** ^1^Department of Psychology, University of Richmond, Richmond, VA, United States; ^2^Department of Psychology, Hampshire College, Hampshire, MA, United States; ^3^Department of Psychology, Claremont McKenna College, Claremont, CA, United States

**Keywords:** undergraduate, collaborative model, course design, publication, cognitive neuroscience

The value of authentic research experience to undergraduates is well-established (Seymour et al., 2004; Lopatto, 2007). These benefits are stronger when associated with conference presentations and published articles (Russell et al., 2007). In addition to advancing knowledge, undergraduate publications are associated with improved writing skills, success in graduate and job applications, clarification of career choice, and are positively associated with continued productivity (Russell et al., 2007; Salsman et al., 2013; Yaffe et al., 2014; Anderson et al., 2015). Publishing with undergraduates benefits both teaching and research programs of faculty mentors (Petrella and Jung, 2008). Despite the benefits of undergraduate publication, faculty continue to face many challenges in providing undergraduates with substantive experiences worthy of publication and in guiding them through the final stage to peer-reviewed publication. Publishing with undergraduate co-authors is particularly challenging for disciplines such as cognitive neuroscience that require complex technologies, multistep data processing, and an understanding of advanced interdisciplinary concepts.

Cognitive neuroscience relates cognitive processes to brain activity and integrates knowledge and skills from many fields. Cognitive electrophysiology (electroencephalography/EEG and event-related potential/ERP) is particularly well-suited to undergraduate education because it is a direct measure of human brain activity that corresponds to cognitive processing in real time, it is relatively inexpensive compared to other cognitive neuroscience methods, and the skills are transferable to many areas of research and to practical applications of science in medicine, engineering, law, etc.

Despite the advantages of cognitive electrophysiology for undergraduate education and its contemporary relevance, few opportunities exist for undergraduates to gain the type of research experience in an EEG/ERP lab that would enable them to publish. Meaningful research experience requires an understanding of experimental techniques and background knowledge to formulate research questions, develop a research plan, collect, analyze and interpret data (Edelson et al., 1999). Moreover, in-depth understanding and exposure to all aspects of the research project are necessary to participate in the dissemination phase of research (VanderStoep and Trent-Brown, 2012). Unfortunately, multiple surveys show that many undergraduate research experiences primarily develop data collection skills and, as a result, students lack the deeper understanding of research design and interpretation necessary to publish results (for a recent review, see Linn et al., 2015). This is especially true for EEG/ERP labs, where student involvement is typically limited to capping and, if they are lucky, cursory preprocessing steps such as eye-blink detection. *If undergraduates lack a conceptual understanding of experimental design, data analysis and interpretation, they cannot be expected to contribute substantively to a peer-reviewed paper*. Table 1 lists knowledge and skills necessary for undergraduates to co-author an ERP publication, divided into three units that can be taught serially.

**Table 1:** Knowledge and skills necessary for undergraduates to co-author an ERP publication.

**General Knowledge**	**Study-specific Knowledge**	**Skills**
**DESIGNING AND CONDUCTING AN ERP EXPERIMENT**		
What ERPs measure and what they reveal about cognitive processing	The specific research question and how it relates to prior knowledge	How to read an ERP paper and conduct a literature search
The nature of the ERP component of interest and its associated cognitive processes	The research hypothesis and how it answers the research question	How to formulate a research hypothesis
Experimental design principles	Elements of the specific experiment design	How to collect ERP data
**ANALYZING ERP DATA**		
The function of each pre-processing stage (baseline correction, filtering, re-referencing epoching, binning, artifact rejection, artifact correction, and averaging)	What processing decisions are appropriate for the study	How to use software to preprocess ERP
Basic statistical knowledge	Which statistical tests are appropriate for the study	How to use statistical software and interpret the output
What differences in amplitudes, latencies and topography mean	The interpretation of the results of the study	How to create and explain ERP figures
**WRITING AN ERP PAPER**		
APA style formatting	A coherent story for the study	How to write clearly and concisely

*General knowledge refers to knowledge that will generalize across any ERP study. Study-specific knowledge refers to knowledge that relates to the particular study that the student is running. Skills refer to generalizable abilities that students must develop through hands-on experience and practice. The table is broken into three units that can be taught across different timeframes or levels of curriculum/experience*.

We suggest that the major roadblock to preparing students for publication using cognitive electrophysiology lies in the enormous time and effort needed to create a curriculum that can effectively allow large numbers of students to learn and integrate the many concepts and skills involved in ERP research. The majority of colleges and universities do not offer undergraduates a course in cognitive electrophysiology, and cognitive neuroscience courses often do not include a cognitive electrophysiology lab component (Bukach et al., 2015). As a result, undergraduates have little understanding of experimental design, data analysis, data interpretation, and may struggle to read an electrophysiology paper when they begin their research experience, making it difficult to bring an EEG/ERP project to publication before they graduate. In 2015, we conducted a faculty survey, and of 206 respondents from both research institutions and primarily undergraduate institutions, 86% indicated a “moderate” or “great” need for electrophysiology undergraduate training materials (Bukach et al., 2015). Additional challenges that can hinder undergraduate publications in this field include limited access to participants or equipment, inadequate technology support, and time constraints on research.

## Collaborative Shared-Resource Model: Pursue

We propose that many of the roadblocks to publishing with undergraduates in disciplines such as cognitive neuroscience can be addressed by a collaborative, shared-resource model that includes both cross-institutional faculty collaboration as well as student-faculty collaboration. Opportunities for engaging students in publishable research increase when faculty from diverse institutions share their time, expertise, and resources. Further, student-faculty collaboration provides opportunities for students to develop their skills and knowledge and ensures that training materials are effective and engaging. The benefits of such collaborative models are numerous, but those most relevant to student publication include an increase in student abilities to apply and generalize learning to new problems and solutions, ask good questions, think critically, synthesize information and ideas, and collaborate (Cox, 2004; Nadelson et al., 2013).

As a working example, we describe how our current initiative, Preparing Undergraduates for Research in STEM-related fields Using Electrophysiology (PURSUE), enhances publication opportunities for not only for those directly involved, but also for others who will benefit from the materials and community established by the project. PURSUE was kickstarted by a grant from the Association of Psychological Science and is currently supported by the National Science Foundation's program for *Improving Undergraduate STEM Education: Education and Human Resources (2016)*. The program is led by three principle investigators (co-authors Bukach, Couperus, and Reed) plus six additional faculty from geographically diverse institutions across the US (North, Northeast, South, Midwest, and West) with disparate student body sizes (1,300–10,200 students), demographics (50–100% female; 11–84% acceptance rate selectivity; 14–61% students of color/international), and interests (applied career focus to experiential focus). The goal of PURSUE is to disseminate and implement best practices in cognitive electrophysiology education for undergraduates with the aim of increasing the quality and number of training opportunities for undergraduates, and increasing research outcomes that involve undergraduate co-authors.

## Critical Components of Pursue

A student-centered semester-long course using evidence-based pedagogy provides undergraduates with a strong conceptual understanding of how EEG/ERP methodology can be used to test theoretical questions, the ability to read original research articles, and the practical knowledge of EEG/ERP experimental design. The addition of lab components that cover data preprocessing, analysis, and interpretation enables students to understand the rationale behind the various choices they must make during data processing and apply their knowledge to interpret novel data. PURSUE incorporates backward course design principles (Wiggins and McTighe, 2005) to first identify the necessary learning outcomes and assessments, and then combine our ideas and best practices to create an engaging and effective set of course materials. Our process involves a continuous cycle of innovation (American Society for Engineering Education., 2009) whereby materials are created, implemented, assessed and revised. The inclusion of faculty from a diverse set of institutions and research areas ensures that the materials are accurate, effective across a variety of contexts, and can be implemented in a flexible manner. The collaborative approach distributes the workload and enhances the quality and creativity of the materials. Undergraduates also contribute to the design, creation, and testing of the materials. Student collaboration ensures that materials are engaging, appropriately scaffolded, and target concepts that are most problematic. Students benefit by developing professional skills and by disseminating the results. Sample undergraduate tutorial videos, animations and interactive simulations can be found on pursueerp.com. Undergraduates conducted experiments to test material efficacy and presented their findings at national conferences (Hagen et al., 2018; Jackson et al., 2018), and are now preparing manuscripts for peer-review. Once course materials have undergone a broader controlled implementation and revision cycle they will be freely available on the Pursue.com website.

A shared database of core ERP experiments and individual difference measures enhances publishing opportunities for undergraduates by reducing the time and resources required to design, program, and conduct an experiment and increases the size and diversity of the sample. EEG/ERP studies often take 2–3 h per participant. Undergraduates have limited time in the lab and may graduate before their project is complete. Further, primarily undergraduate institutions may have limited access to EEG/ERP equipment or subject pools. The PURSUE database is composed of six classic ERP experiments yielding seven ERP components (http://www.erpinfo.org/erp-core) and a rich set of individual difference measures. The database will be used for lab exercises and allows our undergraduates to explore authentic research questions and publish their findings. The three PIs worked collaboratively with undergraduates at every stage of the project. The collaborative model allowed faculty to consult one another on technical issues and share best practices that enhanced the quality and ease of data collection and analysis. Moreover, although still in process, the database has already provided rich opportunities for undergraduate publication: this year 20 undergraduates presented preliminary findings at seven national conferences, four additional undergraduate-led EEG experiments are now in various stages of completion, and one manuscript is submitted for peer-review. As our work shifts to data analysis, the database will provide additional opportunities for undergraduate publication at conferences and in peer-reviewed journals.

A website (pursueerp.com) will freely disseminate our materials, share resources, and build community to expand opportunities for undergraduates beyond PURSUE. The website will facilitate undergraduate publications in cognitive neuroscience by hosting the PURSUE training materials and other resources such as EEG/ERP readings for undergraduates, tips for setting up and running an undergraduate EEG lab, tutorial videos, links to sample experiments, and tips for publishing with undergraduates. We note that PURSUE is still in the design phase, and encourage readers to watch our website for new materials.

A faculty community increases faculty support and opportunities for cross-institutional collaboration. PURSUE faculty participants not only work collaboratively to create training materials and build a database, we also share teaching, procedural, technical and publishing advice, and provide emotional and practical support. Consistent with prior research, PURSUE faculty participants report that their experience provided a sense of community, met needs for academic mentorship, increased motivation to improve their courses, and invigorated their enthusiasm for working with undergraduates in research. It also generated new research collaborations among the members.

## Advantages of Pursue Model

The PURSUE collaborative model can be adopted to facilitate undergraduate opportunities for publication in other academic fields. Because we are geographically dispersed, the PURSUE team meets regularly online, but we have found that in-person meetings at conferences and the occasional local workshops are also crucial to making progress and building community. A “divide and conquer” strategy of forming subgroups also helps to constrain and focus the work. Student-faculty collaboration is slower-paced, due to undergraduate schedules and time necessary for students to develop skills. Team meetings, time management software, and weekly goal-setting help to manage these challenges. We found converting the data collection and analysis protocols to an online survey format helped to guide and document student work. Additionally, setting publication goals for different aspects of the project that are within the timeframe of undergraduate activities is critical. For example, early work on simulations and database collection have been presented at conferences soon after involvement in the work (i.e., within a year).

The advantages of PURSUE's collaborative model for engaging students in publishable research is also perceived by our students. Undergraduates report that the PURSUE experience is unique among other undergraduate research experiences as it allows for direct involvement in every step of the research process, from setting up EEG equipment, establishing a standardized experimental protocol, designing tasks, collecting and analyzing data, and preparing a manuscript. This direct involvement gives them the skills to design and carry out their own ERP studies, as well as a greater understanding of what it means to pursue Cognitive Neuroscience research post-graduation. They find that collaborating with faculty to conduct ERP studies gives them an understanding of the publishing process, and develops the ability to present research topics on a poster and convey information in a concise and meaningful way. Undergraduates also report that with guidance, they learn the nuances of manuscript submission and publication: what is and is not important to include in the methods section of an academic paper, how to form a logical cohesive story with data, and how to speak to the future directions of the conducted research.

## Conclusion

Engaging undergraduates in publishable research necessitates adequate training and resources. A collaborative model in which students and faculty from multiple institutions work together and share their resources helps lead both faculty and students to publishable research outcomes.

## Author Contributions

The model and work described herein was developed and carried out collaboratively by CB, JC, and CR. CB wrote the first draft of the manuscript. KS wrote sections of the manuscript. JC and CR provided comments for revision.

### Conflict of Interest Statement

The authors declare that the research was conducted in the absence of any commercial or financial relationships that could be construed as a potential conflict of interest.
